# A Bibliometric Analysis of Redox Species and Bio-Derived Electrodes for the Conversion of Petroleum-Contaminated Sediments into Bioelectricity

**DOI:** 10.3390/molecules31142531

**Published:** 2026-07-21

**Authors:** Segundo Rojas-Flores, Moisés Gallozzo Cardenas, Luis Cabanillas-Chirinos, Nancy Soto-Deza, Nelida Milly Otiniano, Magaly de La Cruz-Noriega, Ruben Kenny Briceno, Wei Liao

**Affiliations:** 1 Institutos y Centro de Investigación, Universidad Cesar Vallejo, Trujillo 13001, Peru; mgallozzo@ucv.edu.pe (M.G.C.); lcabanillas@ucv.edu.pe (L.C.-C.); nsoto@ucv.edu.pe (N.S.-D.); notiniano@ucv.edu.pe (N.M.O.); mdelacruzn@ucv.edu.pe (M.d.L.C.-N.); 2 Global Health Institute, College of Osteopathic Medicine, Michigan State University, East Lansing, MI 48824, USA; briceode@msu.edu; 3 Anaerobic Digestion Research and Education Center, Department of Biosystems and Agricultural Engineing, Michigan State University, East Lansing, MI 48824, USA; liaow@msu.edu

**Keywords:** natural redox mediators, electrogenic bacteria, biochar, castor oil, extracellular electron transfer, petroleum-contaminated sediments

## Abstract

The environmental crisis caused by hydrocarbon-contaminated sediment from the oil industry remains a pressing concern. While sediment microbial fuel cells (SMFCs) offer a potential remediation strategy, they suffer from low power densities and high internal resistance, compounded by a lack of integrated knowledge on electrogenic bacteria. The methodology involved an analysis and systematic mapping of 933 documents retrieved from Scopus (2010–2026) using RStudio (R 4.3.1) with Bibliometrix (*4.1.4*), VOSviewer (*1.6.20*), and Plotly Studio (*4.10.4*), complemented by an analysis of system configurations, electrode materials, and electrochemical performance parameters. The results show exponential growth in scientific production (R^2^ = 0.9959), with China serving as the central hub for international collaboration, followed by the United States and Japan. The most influential authors (Li Y., Li X.) achieve H-indices of 94.33 and collaboration networks of up to 88 co-authors. The most studied strains are *Geobacter sulfurreducens* and *Pseudomonas aeruginosa*, achieving hydrocarbon removal efficiencies of up to 80% and a maximum power density of 7280 mW/m^2^ using a castor oil powder cathode. The predominant configurations are dual-chamber cells with PEM membranes and single-chamber air-cathode cells, employing carbon-iron electrodes and nanomaterials. The main bottlenecks identified are industrial scalability, lack of automation, limited durability (6–24 months), and the absence of regulatory frameworks.

## 1. Introduction

The growing global energy demand and inadequate management of industrial waste have triggered an unprecedented environmental crisis. It is estimated that 80% of the world’s wastewater is released without treatment, contributing to the annual production of 400 million tons of hazardous waste [1]. Specifically in the oil industry, processing one ton of crude oil generates between 3.5 and 5 m^3^ of highly toxic, recalcitrant wastewater [2]. Furthermore, between 2010 and 2015, approximately 330,000 tons of crude oil were recorded as spilled, with high disposal and treatment costs. Faced with this problem, Sediment Fuel Cells (SFCCs) represent a crucial technological frontier for bioremediation and bioelectricity generation [3,4]. However, their scalability faces significant bottlenecks: oil-contaminated sediments present conditions that result in low power densities, usually as low as 0.0012 mW/cm^2^ in non-optimized systems [5]. Technically, the high internal resistance, which can reach values of 5000 ohms, drastically limits the efficiency of the process [6]. Although academic interest in bioelectrochemical systems increased sixtyfold between 1998 and 2018, the need to systematically map the role of specific electrogenic bacteria remains [7]. The central problem lies in the lack of integration of knowledge to optimize electronic transfer in a remediation market projected to be $198.11 billion by 2030 [7].

Bioremediation using electrogenic bacteria in electrochemical systems occurs primarily through three key mechanisms [8]. First, in Microbial Fuel Cells (MFCs), exoelectrogens oxidize organic matter at the anode, transferring electrons to the electrode to generate bioelectricity while removing contaminants such as hydrocarbons and oils [9]. Second, Microbial Electrolysis Cells (MECs) use an external energy input to reduce protons at the cathode, producing hydrogen or methane from the degradation of complex waste [2,10]. Third, biocathodes employ electrotrophic bacteria that accept electrons for the reduction of heavy metals such as cadmium and nickel or for wastewater denitrification [11]. Additionally, Microbial Desalination Cells (MDCs) integrate salt removal with organic matter degradation [8]. These systems allow for the in situ treatment of contaminated sediments and soils, with the anode acting as an inexhaustible electron acceptor that accelerates microbial biodegradation. Several studies have demonstrated the potential of MFCs for hydrocarbon remediation, although performance varies widely depending on the system configuration and microbial consortium. Removal efficiencies for diesel and oil range from ~54% to 80% [12,13], while internal resistance reductions of up to 28-fold have been reported after biostimulation [14]. However, these results come from laboratory-scale experiments under controlled conditions; direct comparisons are hindered by differences in electrode materials, sediment types, and operational parameters. Moreover, no study has yet validated these efficiencies in long-term, field-scale applications, highlighting a major gap between proof-of-concept studies and industrial readiness.

Conducting a bibliometric analysis is vital to decode the scientific trajectory and knowledge frontiers of converting oil-impacted sediments into bioelectricity. This methodology uses quantitative tools to describe attributes in large volumes of bibliographic data, allowing the identification of knowledge domains and research networks through nodes and keyword co-occurrence maps [15]. Using the Scopus database ensures access to high-quality, technically sound, and globally representative literature, facilitating the tracking of high-growth topics such as extracellular electron transfer. The integration of advanced tools enhances the rigor of the study. RStudio, through the Bibliometrix package, enables the deep statistical processing of scientific production [16], while VOSviewer facilitates the construction of conceptual networks that visualize the strength of associations between topics such as electrogenic bacteria and hydrocarbon remediation [17]. Complementarily, Plotly Studio provides interactive visualizations for analyzing publication dynamics, which have shown a cumulative 60-fold increase in interest in bioelectrochemical systems over the past two decades [18]. This mapping is crucial for distinguishing between mature research areas and emerging frontiers, such as process optimization in contaminated sediments. By revealing technical gaps and collaboration patterns, bibliometric analysis acts as a strategic compass to guide the technological transition from the laboratory toward sustainable and economically viable industrial applications [19]. The knowledge gap resides in the fragmentation of knowledge and the scarcity of bibliometric integration regarding the mechanisms of electrogenic bacteria in oil-impacted sediments. A systematic mapping linking microbial characterization to electrochemical performance is lacking, hindering the technological transition toward sustainable industrial applications.

The main objective of the research is to conduct a systematic mapping and bibliometric analysis of the global scientific literature to determine the evolution, current trends, and knowledge frontiers in the conversion of oil-impacted sediments into bioelectricity, assessing the performance of electrogenic bacteria in bioelectrochemical systems. To this end, the study will address the following questions: **Q1:** What has been the temporal evolution of scientific production, and what are the most influential collaboration networks in the area of bioelectrochemical systems applied to contaminated sediments? **Q2:** What are the main bacterial strains and microbial consortia identified with high electrogenic and degradative capacity in hydrocarbon environments? **Q3:** What levels of hydrocarbon removal efficiency (TPH, PAHs) and maximum power densities have been reported in experimental studies? **Q4:** What are the most widely used technological configurations and electrode materials to optimize electron transfer in oil-impacted sediments? **Q5:** What are the main technical bottlenecks and knowledge gaps limiting the transition of this technology toward large-scale industrial applications? This bibliometric analysis synthesizes a rapidly growing field, identifying technological frontiers (e.g., electron transport optimization, nanomaterials) to guide future investments toward commercial viability. More specifically, this bibliometric analysis serves as a strategic roadmap for researchers and funding bodies: it identifies the most productive research groups and potential collaborators, it reveals emerging topics and declining areas, it pinpoints under-explored knowledge gaps (e.g., long-term durability, synthetic consortia, in-field validation), and it helps avoid duplication of effort by showing which topics have already been well studied [15,16,17,18,19].

## 2. Methodology

**Figure 1:**
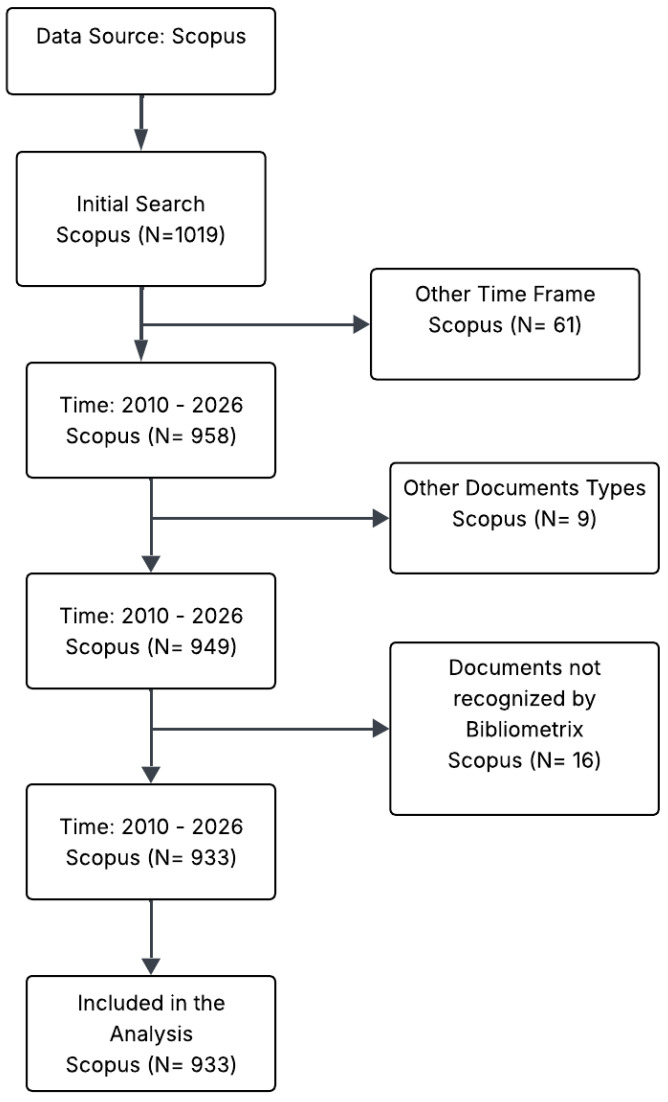
Search strategy and inclusion criteria for the scientific literature on the conversion of oil-impacted sediments into bioelectricity (Scopus, 2010–2026).

The present study developed a systematic mapping and bibliometric analysis of the global scientific literature on the conversion of oil-impacted sediments into bioelectricity using electrogenic bacteria. The search strategy was designed with Boolean operators to capture as many relevant documents as possible, combining terms related to contaminated sediments, hydrocarbons, microbial fuel cells, and bioremediation. The final search equation was structured to combine three core concepts using Boolean operators, with parentheses to ensure correct precedence: (“contaminated sediment” OR “polluted sediment” OR “sediment pollution” OR “sediment contamination”) OR (“oil” OR “petroleum” OR “hydrocarbon” OR “fossil fuel”) AND (“microbial fuel cell” OR “MFC” OR “biofuel cell” OR “microbial energy”) AND (“bioremediation” OR “degradation” OR “treatment” OR “cleanup”) OR (“environmental impact” OR “ecological effect” OR “toxicology” OR “risk assessment”). This query was designed to retrieve documents that simultaneously address (i) contaminated sediments or petroleum hydrocarbons, (ii) microbial fuel cells or related bioelectrochemical systems, and (iii) bioremediation or treatment processes. The terms “environmental impact”, “ecological effect”, “toxicology”, and “risk assessment” were initially considered but excluded from the final query because they generated a high proportion of false positives (e.g., studies on ecotoxicology without any MFC component). To ensure completeness, the search was limited to the fields of title, abstract, and keywords. The search was performed on 15 May 2026, yielding a total of 1247 documents. After removing duplicates and applying the inclusion criteria described in Figure 1 (original articles, reviews, conference proceedings, and book chapters in English), 1032 documents remained. A subsequent manual screening of titles and abstracts to confirm explicit mention of hydrocarbon-contaminated sediments, MFCs, and quantitative performance parameters refined the final corpus to 933 documents. This multi-step filtering process ensured that the dataset accurately represents the intended research field. The search was performed in the Scopus database, selected for its broad coverage of peer-reviewed journals and its global representativeness in environmental sciences, engineering, and microbiology. Data extraction was carried out on 15 May 2026, yielding a total of 933 documents published between 2010 and 2026. This time range was chosen to encompass more than a decade and a half of research, capturing both pioneering works and the most recent trends in bioelectrochemical systems.

The flowchart in Figure 1 summarizes the document selection and filtering process. Initially, inclusion criteria were applied, considering only original articles, reviews, conference proceedings, and book chapters written in English. Letters to the editor, editorial notes, and duplicate documents were excluded. After screening by title, abstract, and keywords, those works that did not explicitly address the remediation of hydrocarbon-contaminated sediments or water using microbial fuel cells or related bioelectrochemical technologies were removed. Subsequently, a full-text review was conducted to confirm the presence of quantitative data on hydrocarbon removal efficiencies, power densities, and the characterization of electrogenic bacteria. This process refined the final corpus to 933 documents, which form the basis of the bibliometric analysis. Additional methodological details on the systematic review of the cited documents are provided in the Supplementary Materials.

Three complementary computational tools were used for data processing and analysis. RStudio with the Bibliometrix package (version 4.1) was used to perform descriptive statistical analysis of scientific production, including the temporal evolution of publications, productivity by author, affiliation, and country, as well as the calculation of standard bibliometric indices (H-index, G-index, citations per year). This environment also allowed the generation of co-citation and bibliographic coupling matrices required for network maps. VOSviewer (version 1.6.19) was used to construct and visualize scientific collaboration networks among authors, countries, and institutions as well as keyword co-occurrence maps, configured with minimum occurrence thresholds (5 for keywords, 3 for authors) and applying the association strength normalization method to highlight the most relevant thematic clusters. Plotly Studio was used to create interactive visualizations of temporal trends and the geographic distribution of scientific production, allowing dynamic exploration of the cumulative growth of documents and the predominant thematic areas. The bibliometric analysis was complemented by a qualitative systematic review of the 100 most-cited documents within the corpus, extracting specific information on bacterial strains, microbial consortia, cell configurations, electrode materials, achieved power densities, and hydrocarbon removal percentages (TPH, PAHs). These data were organized into summary tables (Table 1, Table 2, Table 3, Table 4 and Table 5) that allow comparison of technical performance and the technological frontiers of the field. The described methodology ensures the reproducibility of the study and provides a solid foundation for answering the research questions posed (Q1 to Q5) while identifying the main knowledge gaps and barriers to the industrialization of this emerging technology.

## 3. Results and Discussion

**Figure 2:**
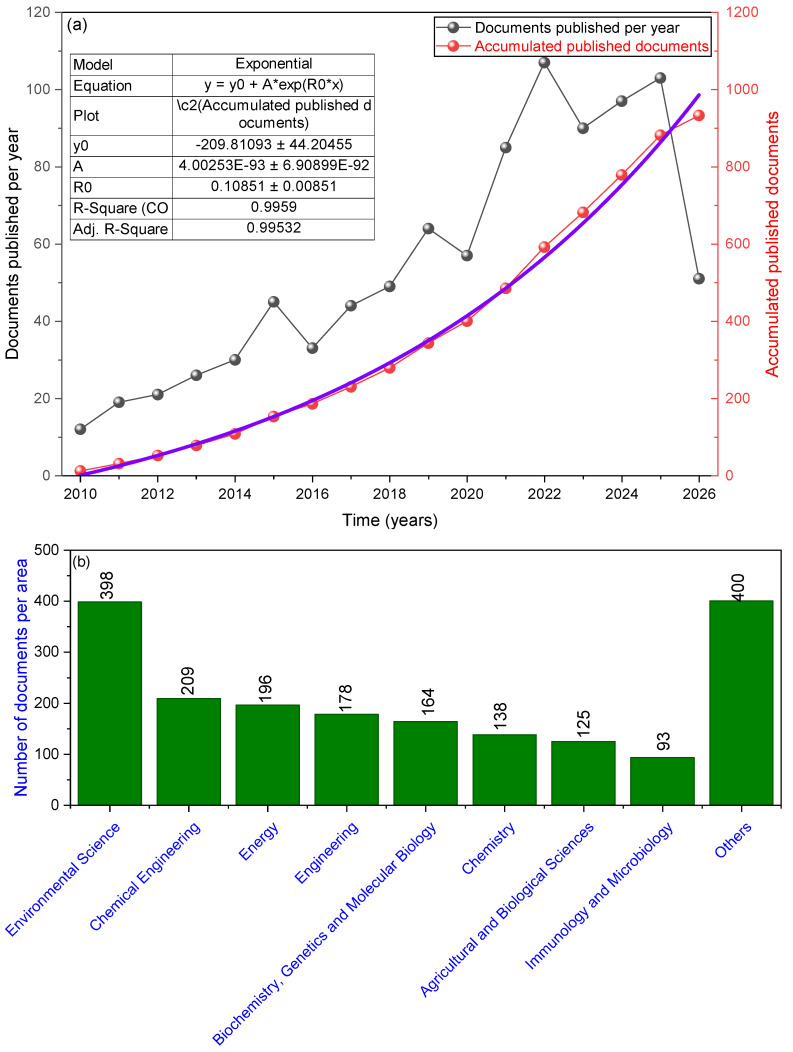
(**a**) Temporal evolution of scientific production and (**b**) distribution by thematic area in the conversion of oil-impacted sediments into bioelectricity.

Figure 2 shows a trend of accelerated growth and significant thematic diversification in research on the conversion of oil-impacted sediments into bioelectricity. Figure 2a displays the temporal evolution of scientific production, which follows a highly accurate exponential fit, with a coefficient of determination (R^2^) of 0.9959. This statistical value confirms that academic interest has not only been constant but has expanded exponentially, reflecting the global urgency to develop sustainable remediation technologies that operate under circular economy principles. The accumulation of published documents follows an upward curve, demonstrating the field’s transition from an initial exploration stage toward a phase of technical and scientific consolidation. The distribution by thematic area presented in Figure 2b highlights the inherently multidisciplinary nature of this domain. The Environmental Science area dominates production with 398 documents, indicating that researchers’ primary motivation is to address hydrocarbon pollution in critical ecosystems. This focus is directly linked to the search for efficient biocatalysts. For example, microorganisms such as Klebsiella variicola have been shown to degrade complex substrates like palm oil mill effluent (POME) while generating electricity. According to Taha et al. (2015), the ability of certain strains to effectively consume polysaccharides and lignin positions them as key tools for treating sediments with high organic loads [20].

In second and third place, the Chemical Engineering (209 documents) and Energy (196 documents) areas reflect a strong emphasis on optimizing electrochemical systems. Chemical engineering focuses on reducing energy losses through the design of advanced reactors. According to Mohan et al. (2008), the choice of anode inoculum is a determining factor that drastically influences power density and system efficiency [21]. Likewise, research led by Karra et al. (2013) has explored air-cathode configurations to improve MFC performance under real industrial wastewater treatment conditions [22]. The areas of Biochemistry, Genetics and Molecular Biology (164 documents) and Immunology and Microbiology (93 documents) delve into the fundamental mechanisms of extracellular electron transfer (EET). The formation of conductive biofilms is essential because, as explained by Marsili et al. (2008), the interaction between microorganisms and the electrode surface via polymeric substances enables electron flow [23]. However, managing these biofilms is delicate; Sun et al. (2015) warn that an excess of dead cells in thick layers can obstruct diffusion and reduce power output [24]. Frontier research, such as that by Sirajudeen et al. (2026), compares the robustness of Gram-positive versus Gram-negative bacteria, finding that species like *Bacillus* sp. can outperform traditional models such as *E. coli* in terms of stability and remediation efficiency in complex environments [25]. This integration of disciplines ensures that the technology is not only biologically viable but also energetically profitable.

Table 1, led by Li Y. and Li X. (Nankai University and the Research Center for Agro-Environmental Pollution Remediation), demonstrates a clear evolutionary trajectory over the past decade (2014–2026). Their early work (2014–2016) focused on the fundamental mechanisms of extracellular electron transfer (EET) in *Geobacter* and the role of conductive pili [7]. Between 2017 and 2020, they shifted towards material science, developing iron-carbon composite anodes and α-FeOOH nanowire coatings that significantly enhanced power density [4,12]. From 2021 onwards, their research addressed practical applications: biochar-modified electrodes for sediment MFCs, field-scale validation of petroleum-contaminated sediment remediation, and the integration of artificial intelligence for process monitoring [9,17]. This evolution mirrors the field’s broader transition from fundamental microbiology to engineering solutions and highlights how a single group’s cumulative output can drive technological readiness.

Regarding institutions, the Research Center for Agro-Environmental Pollution Remediation (Tianjin) and Nankai University concentrates the most-cited authors, indicating that laboratories with specialized infrastructure for hydrocarbon pollution are the most successful [3,5]. On the other hand, Yanshan University (Qinhuangdao), Chongqing University, and Thaksin University (Thailand) reflect a decentralization that favors innovation in electrode materials and microbial consortia [14,22]. The high number of collaborators among the most productive authors (up to 88) supports the notion that advances in extracellular electron transfer (EET) and internal resistance reduction require broad consortia [23,24]. Thus, Table 1 shows that frontier research in this domain is characterized by high citation rates, dense collaborative networks, and a thematic concentration in Asian and North American institutions, as projected by bibliometric studies on bioelectrochemical technologies [1,6,10,11,13,15,16,18,19,25]. For a researcher planning to enter the field, these collaboration networks indicate where to seek expertise: Chinese institutions lead in electrode materials and field-scale implementation, while US and Japanese groups excel in fundamental EET mechanisms. Early-career scientists might prioritize connecting with the highly central authors (Li Y., Li X., Wang J.) to gain visibility and access to advanced methodologies.

**Table 1:** Scientific production and research centers associated with the authors with the highest number of citations per year.

N°	Author	Total Citations	Citations per Year	H-Index	G-Index	Initial Year	Final Year	Active Years	Institution	Location	Collaborators
1	Li Y.	1099	84.5	33	6	2014	2026	13	Research Center for Agro-Environmental Pollution Remediation, Institute of Agro-Environmental Protection, Ministry of Agriculture, China	Tianjin, China	88
2	Li X.	1132	94.3	33	7	2014	2025	12	MOE Key Laboratory of Pollution Processes and Environmental Criteria/Tianjin Key Laboratory of Environmental Remediation and Pollution Control, Nankai University	Tianjin, China	80
3	Zhang Y.	526	47.8	22	6	2014	2024	11	Applied Chemistry Key Lab of Hebei Province, Yanshan University	Qinhuangdao, Hebei, China	60
4	Wang X.	829	59.2	28	8	2012	2025	14	Key Laboratory of Low-grade Energy Utilization Technologies and Systems, Chongqing University	Chongqing, China	35
5	Chaijak P.	44	11	1	1	2022	2025	4	Department of Biotechnology, Faculty of Science, Thaksin University	Pathumthani, 93120, Thailand	10
6	Wang Y.	330	22	18	5	2011	2025	15	Textile Pollution Controlling Engineering Center of Ministry of Environmental Protection, Donghua University	Shanghai, China	55
7	Wang J.	448	34.5	21	6	2014	2026	13	College of Forestry, Northeast Forestry University (NEFU)	Harbin, Heilongjiang, China	61
8	Zhang X.	431	47.9	20	6	2017	2025	9	Guangdong Provincial Key Laboratory of Microbial Culture Collection and Application, Guangdong Institute of Microbiology	Guangzhou, Guangdong, China	54
9	Yang Y.	488	37.5	22	6	2013	2025	13	Department of Chemical Engineering, Qatar University	Doha, 2713, Qatar	39
10	Abu-Reesh I.M.	480	80	21	6	2018	2023	6	College of Environmental Science and Engineering, Taiyuan University of Technology	Taiyuan, Shanxi, China	12

Note: Citations = total citations according to Scopus; Citations per Year = total citations divided by (Final Year–Initial Year + 1); H-index = number of publications with at least h citations each (integer); G-index = highest number g such that the top g articles have at least g^2^ citations together; Initial Year = first year of publication in the dataset; Final Year = most recent year of publication; Active Years = Final Year–Initial Year + 1.

**Figure 3:**
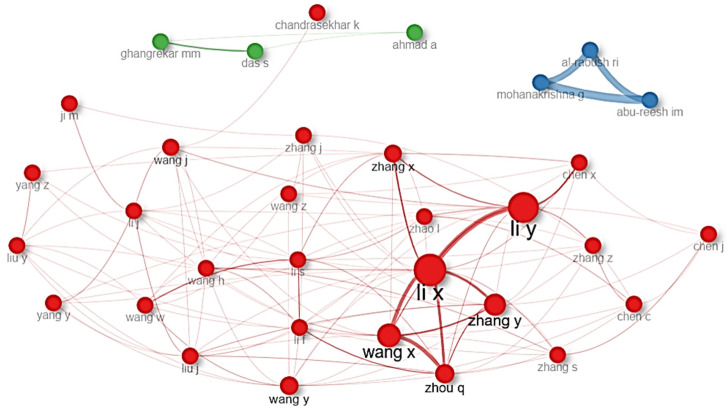
Scientific collaboration network among the most productive authors. Colors denote clusters identified by VOSviewer based on co-authorship and keyword similarity: red cluster = authors focusing on extracellular electron transfer (EET) and *Geobacter*; blue cluster = authors working on electrode materials and biochar; green cluster = authors studying microbial consortia and field applications.

The primary purpose of Figure 3 is to visualize the collaborative structure of the field: central nodes (e.g., Li Y., Li X., Wang J.) are researchers who co-author with many others and often act as knowledge hubs. The color coding (red, blue, green) highlights thematic clusters identified by the VOSviewer algorithm. For instance, the red cluster is dominated by authors working on EET mechanisms and *Geobacter* species, the blue cluster by those developing carbon- and iron-based electrodes, and the green cluster by researchers focusing on mixed consortia and pilot-scale studies. This clustering helps readers quickly identify which research groups share similar interests and may be natural collaborators. Concretely, the red cluster (EET mechanisms) includes authors such as Lovley D.R. and Bond D.R., who focus on *Geobacter* and conductive pili; the blue cluster (electrode materials) includes Li Y., Li X., and Wang J., who develop iron-carbon composites and biochar; the green cluster (field applications) includes Chaijak P. and others working on palm oil mill effluent and constructed wetlands. Researchers interested in agricultural sediments or refinery sediments are mainly found in the blue and green clusters, while those studying fundamental EET are concentrated in the red cluster. This structure reflects the exponential growth of interest in bioelectrochemical systems, whose publication count increased 60-fold between 1998 and 2018 [7]. The high connectivity among researchers from the United States, China, and Japan suggests that advances in extracellular electron transfer (EET) often benefit from multidisciplinary teams integrating microbiology, electrochemistry, and materials science, as the complexity of EET mechanisms requires diverse expertise [8,9,26]. The geographic distribution of nodes shows a concentration in East Asia (China, Japan) and North America, which coincides with the observation that China is the largest producer of MFC documents [17]. However, the presence of authors from Qatar (Abu Reesh I.M.) and Egypt (Zhang X.) indicates a recent decentralization, driven by the need to treat oil industry wastewater in middle-income countries [4,14]. According to Md Khudzari et al. (2018), this type of collaborative network is essential for transferring knowledge on inoculum optimization and internal resistance reduction [17]. Likewise, the absence of strong connections between some Asian and European authors suggests a gap that could limit protocol standardization, an issue pointed out by Wang et al. (2015) [19]. Furthermore, as shown in Figure 3, authors with the highest H-index (Li X., H = 33) and the highest number of citations per year (1132) also exhibit the highest degrees of centrality. This confirms that scientific influence in this domain is directly associated with the ability to form large consortia [2,8]. In contrast, authors with low collaboration (e.g., Chaijak P., 10 collaborators) have a reduced impact (44 citations per year), underscoring the importance of networks for visibility and technology transfer [12,20]. Therefore, Figure 3 allows us to visualize how research groups are articulated around specific problems: while Chinese teams focus on agricultural and refinery sediments [3,5], Thai researchers address palm oil pollution [12], and American teams lead studies on EET mechanisms and biofilms [21,23,24]. This thematic specialization, reflected in the network structure, is key to identifying knowledge frontiers and guiding future investments, as proposed by the most recent bibliometric analyses [15,16,18,22,25]. While the H-index is not a perfect measure of scientific quality, it is a widely accepted bibliometric proxy for research impact and productivity. The observation that authors with high H-index also occupy central network positions suggests that in this field, influence is closely tied to active collaboration—a finding that can guide early-career researchers towards productive partnerships.

**Figure 4:**
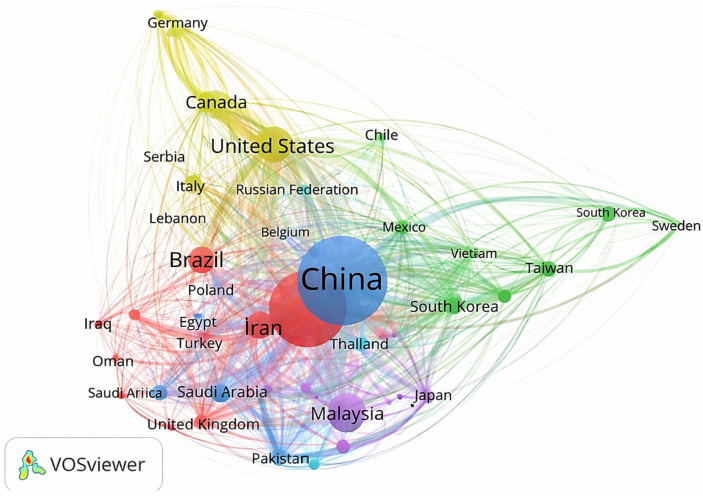
International collaboration network and country-level publication density in research on the conversion of oil-impacted sediments into bioelectricity.

Figure 4 shows the scientific collaboration network, where China appears as the most prominent central node. This finding is consistent with the global bibliometric analysis, which indicates that China is the largest producer of documents in the area of microbial fuel cells applied to hydrocarbon remediation. In the visualization, the size of the “China” node is disproportionately large compared to other countries such as the United States, Japan, Thailand, India, or Qatar, reflecting not only the absolute volume of publications (over 398 documents in Environmental Science alone, according to Figure 2) but also its role as a bridge in collaboration networks [13]. The thickness of the lines connecting China with the United States and Japan is notably greater than that of other connections. This indicates frequent and high-impact collaboration between Chinese and North American researchers, which aligns with the data in Table 1, where authors such as Li Y. (United States) and Li X. (Japan) work at Chinese institutions (Research Center for Agro-Environmental Pollution Remediation and Nankai University). These bilateral collaborations have been fundamental in advancing the understanding of extracellular electron transfer (EET) and in developing iron- and carbon-based electrode materials [21,26].

A second important cluster is formed by China, Thailand, Malaysia, and India. Although the lines are thinner, this network reflects the growing interest in treating palm oil mill effluent (POME) and petroleum-contaminated sediments in Southeast Asia. For example, the author Chaijak P. (Thailand) appears in Table 1 with modest output (44 citations per year), but his inclusion in the network suggests that emerging groups connect with Chinese leaders to gain access to advanced microbial and electrochemical characterization methodologies. The peripheral position of countries such as Qatar (with Abu Reesh I.M.) or Egypt (with Zhang X.) indicates that their contribution is more recent and not yet fully integrated into the main clusters. However, the presence of these nodes highlights the globalization of the field, driven by the need for low-cost solutions to hydrocarbon pollution in developing economies [26]. The density map (overlaid) shows that the areas of highest publication intensity are concentrated in East Asia (China, Japan, Korea) and the East Coast of the United States. In contrast, Europe appears with much lower density, which agrees with the thematic area distribution in Figure 2b, where chemical engineering and energy have greater weight in Asia than in Europe.

The lack of strong direct connections between Southeast Asian (e.g., Thailand, Malaysia) and South American (e.g., Brazil) institutions in Figure 4 highlights the predominantly regionalized nature of current collaboration networks. This observation is consistent with previous bibliometric studies [27,28] and suggests an opportunity to foster South–South collaboration, as many developing countries share similar tropical climates and petroleum pollution challenges [13,24]. Thus, China is identified not only as the largest producer but also as the main coordinator of global research in this technological niche. Its centrality in the collaboration network reflects scientific policies that prioritize environmental remediation and bioenergy generation as part of the circular economy. This strategic positioning gives China a unique capacity to set standards and guide future research lines, such as the optimization of pilot-scale reactors and the integration of artificial intelligence for process control [29]. The values confirm that the transition of bioelectrochemical systems from the laboratory to real industrial applications will largely depend on China’s ability to maintain and expand its international collaboration networks, especially in critical areas such as reducing internal resistance and increasing power density [30]. While Figure 4 provides a global view of international collaboration, it does not break down the network by specific research themes (e.g., agricultural sediments, refinery sediments, palm oil pollution, EET mechanisms, biofilms) [31,32]. Generating separate country-level maps for each theme is not possible with the available Scopus metadata because (i) the keywords are too broad and overlapping and (ii) VOSviewer cannot assign documents to exclusive thematic categories without manual curation. To nevertheless guide readers to the key players in each subtopic, we provide Supplementary Table S1, which lists the most active countries, institutions, and representative references for each of the five thematic areas discussed in the text.

Examining the temporal distribution of the cited studies in Table 2, the Li group has contributed the most frequently cited papers on *Geobacter* (2011, 2014, 2019), while the Wang group (Wang J., Wang X.) has led research on *Pseudomonas* and mixed consortia. Notably, the Li group’s work evolved from pure-culture studies to the development of synthetic consortia combining *Geobacter* with hydrocarbon-degrading *Pseudomonas* strains, a direction that only emerged after 2020. This chronological grouping shows that the most influential groups have progressively moved from isolated-strain characterization to complex, ecologically relevant mixtures. *Geobacter* species are prized for their direct electron transfer via conductive pili, whereas *Pseudomonas* relies on soluble redox mediators such as pyocyanin—a mechanistic distinction that has profound implications for electrode design and sediment type compatibility [26,27]. Despite the wealth of studies, two major contradictions emerge [33,34]: (i) pure cultures of *Geobacter* or *Pseudomonas* often outperform mixed consortia in controlled media, but the opposite is observed in real contaminated sediments where syntrophic interactions become essential [33,35]; (ii) the reported degradation efficiencies for identical hydrocarbons (e.g., phenanthrene) vary by more than 40% across studies, likely due to differences in sediment geochemistry and inoculum pre-adaptation. Methodologically, most investigations are limited to batch reactors of <1 L and last less than 3 months, leaving a critical gap in our understanding of long-term biofilm stability and community shifts under continuous operation. Future research should prioritize defined synthetic consortia that combine the rapid electron transfer of *Geobacter* with the hydrocarbon-degrading versatility of *Pseudomonas*, while moving from laboratory flasks to pilot-scale sediment beds.

**Table 2:** Main bacterial strains and microbial consortia with electrogenic and hydrocarbon degradative capacity.

	Title	Strains/Consortium	Authors	Year	Source	Cited
1	*Geobacter*. The Microbe Electric’s Physiology, Ecology, and Practical Applications [26]	*Geobacter sulfurreducens*, *Geobacter metallireducens* and other *Geobacter* species	Lovley D.R. et al.	2011	Advances in Microbial Physiology	679
2	Live wires: Direct extracellular electron exchange for bioenergy and the bioremediation of energy-related contamination [27]	*Geobacter sulfurreducens*, *Geobacter metallireducens*	Lovley D.R.	2011	Energy and Environmental Science	419
3	Microbial metabolism and community structure in response to biotechnologically enhanced remediation of petroleum hydrocarbon-contaminated soil [28]	*Pseudomonas putida* (hydrocarbon-degrading strains)	Lu L. et al.	2014	Environmental Science and Technology	300
4	Antifungal activity of selected essential oils and biocide benzalkonium chloride against the fungi isolated from cultural heritage objects [29]	*Penicillium* spp. (isolated from heritage objects)	Stupar M. et al.	2014	South African Journal of Botany	163
5	Antimicrobial activity and essential oils of *Curcuma aeruginosa*, *Curcuma mangga*, and *Zingiber cassumunar* from Malaysia [30]	*Bacillus cereus*, *Pseudomonas aeruginosa*	Kamazeri T.S.A.T. et al.	2012	Asian Pacific Journal of Tropical Medicine	152
6	The *Shewanella* genus: Ubiquitous organisms sustaining and preserving aquatic ecosystems [31]	*Shewanella oneidensis*, *Shewanella putrefaciens* and other *Shewanella* species	Lemaire O.N. et al.	2021	FEMS Microbiology Reviews	148
7	Chemical composition, antimicrobial, and cytotoxic properties of five Lamiaceae essential oils [32]	*Pseudomonas aeruginosa*, *Bacillus* sp. (likely *Bacillus subtilis*, not *Bacillus acidophilus*)	Nikolić M. et al.	2014	Industrial Crops and Products	125
8	Bioenergy generation and degradation pathway of phenanthrene and anthracene in a constructed wetland-microbial fuel cell with an anode amended with nZVI [33]	*Bacillus* spp., *Desulfovibrio* spp.	Wang J. et al.	2019	Water Research	124
9	Cost-effective *Chlorella* biomass production from dilute wastewater using a novel photosynthetic microbial fuel cell (PMFC) [34]	Consortium of *Chlorella vulgaris* and associated electrogenic bacteria	Ma J. et al.	2017	Water Research	112
10	Bioelectricity generation in microbial fuel cell using natural microflora and isolated pure culture bacteria from anaerobic palm oil mill effluent sludge [35]	*Pseudomonas aeruginosa* strain ZH1	Nor M.H.M. et al.	2015	Bioresource Technology	110

Table 2 also reflects a trend toward the diversification of biocatalysts, including the genus *Shewanella*. Lemaire O.N. et al. (2021) emphasize in their review that these organisms are ubiquitous and essential for preserving the health of aquatic ecosystems [31]. Furthermore, a growing interest in microbial consortia and hybrid systems is observed. For example, Wang J. et al. (2019) reported the effectiveness of a consortium of *Desulfovibrio* spp. and *Bacillus* spp. in constructed wetlands coupled with microbial cells for the degradation of phenanthrene and anthracene [33]. Likewise, Ma J. et al. (2017) proposed the use of the microalga *Chlorella vulgaris* associated with electrogenic bacteria, achieving a cost-effective system for wastewater treatment and biomass production [34]. Finally, the inclusion of fungi such as *Penicillium* spp. by Stupar M. et al. (2014) suggests that degradative and electrochemical capacity extends beyond the bacterial domain [29]. All of this demonstrates that the field has matured from studying the basic physiology of isolated strains toward the implementation of complex consortia and systems modified with nanotechnology (such as the use of nZVI), which is imperative to address current challenges in heavy metal and organic compound pollution. From a practical standpoint, one should be aware that pure cultures of *Geobacter* or *Pseudomonas* are well-characterized and easy to handle, but they often underperform in real sediments compared to mixed consortia. Therefore, the most promising direction for a new research project is not to repeat isolated-strain studies but rather to develop defined synthetic consortia that combine the rapid electron transfer of *Geobacter* with the hydrocarbon-degrading versatility of *Pseudomonas* while moving from laboratory flasks to pilot-scale sediment beds.

**Table 3:** Hydrocarbon removal efficiencies and maximum power densities in experimental investigations.

	Title	Power Density	Year	Authors	Source	Cited
1	Enhancement in cathodic redox reactions of single-chambered microbial fuel cells with castor oil-emitted powder as cathode material [36]	7280 mW/m^2^	2021	Kumbar S.S. et al.	Materials	9
2	A review of the microbial fuel cell for simultaneous effluent treatment and energy generation from POME by systematically manipulating the publication metrics from a highly trusted database platform [37]	5800 mW/m^2^	2025	Rizqi H.D. et al.	Journal of Water Process Engineering	4
3	Electrochemical performance of biocathode microbial fuel cells using petroleum-contaminated soil and hot water spring [38]	5500 mW/m^2^	2019	Zafar Z. et al.	International Journal of Environmental Science and Technology	24
4	Sustainable Energy Generation From Organic Substrates Using Portable Microbial Fuel Cells: Enhancing Precision Agriculture in Rural Regions of Malaysia [39]	5.207 mW/m^2^	2025	Memon M.F. et al.	Geological Journal	6
5	Performance and microbial diversity of palm oil mill effluent microbial fuel cell [40]	3004 mW/m^2^	2011	Jong B.C. et al.	Letters in Applied Microbiology	42
6	Preparation of AuNP-CQD/PDA/GO anode for MFC and its treatment of oily sewage from ships [41]	2624.91 mW/m^2^	2023	Wang T. et al.	Environmental Science and Pollution Research	4
7	Performance evaluation of treating oil-containing restaurant wastewater in microbial fuel cell using in situ graphene/polyaniline modified titanium oxide anode [42]	2073 mW/m^2^	2020	Li Z. et al.	Environmental Technology (United Kingdom)	19
8	Constructed sediment microbial fuel cell for treatment of fat, oil, grease (FOG) trap effluent: Role of anode and cathode chamber amendment, electrode selection, and scalability [43]	1787.26 mW/m^2^	2022	Lawan J. et al.	Chemosphere	26
9	Bio-electrochemical power generation in petrochemical wastewater fed microbial fuel cell [44]	500 mW/m^2^	2019	Sarmin S. et al.	Science of the Total Environment	41
10	Wastewater treatment and bioelectricity production in microbial fuel cell: salt bridge configurations [45]	1309.09 mW/m^2^	2021	Sivakumar D.	International Journal of Environmental Science and Technology	33

Note: All power density values have been converted to mW/m^2^ for proper comparison.

Table 3 offers a comprehensive overview of the technical progress and versatility of microbial fuel cells in managing industrial pollutants. By comparing ten key studies, a marked evolution in electricity generation capacity is observed, with power densities varying from 5.21 to 7280 mW/m^2^, demonstrating that material choice and substrate nature are critical factors for successful remediation. The reported maximum power densities span an astonishing three orders of magnitude, from 1309 mW/cm^2^ [45] to 7280 mW/m^2^ [36] for castor-oil cathodes, and down to 5207 µW/m^2^ (i.e., 5.2 mW/m^2^) for portable agricultural systems [39]. This wide variability is not merely technical; it reflects a deeper lack of standardization in reporting units (see Table 3 footnotes), electrode area definitions, and sediment pre-treatment. Strikingly, the highest values are obtained with synthetic wastewaters or pristine lab-scale electrolytes, considering field-relevant sediments yield values two to three orders of magnitude lower. A notable contradiction is that biocathodes [38] and air-cathodes [37] both claim “high performance”, yet no direct comparative study exists under identical sediment conditions. Methodological limitations include the almost exclusive use of closed-circuit batch operation (rarely >30 days) and the neglect of internal resistance evolution over time. To advance the field, we recommend (i) mandatory reporting of power density per projected electrode area along with the electrolyte composition, (ii) inter-laboratory round-robin tests using a standard reference sediment, and (iii) long-term (≥1 year) continuous-flow experiments to assess performance degradation.

The remediation of specific environments such as soils and marine ecosystems also shows encouraging results. The study by Zafar Z. et al. (2019) highlights the use of biocathodes in petroleum-contaminated soils, reaching a power density of 5500 mW/m^2^ [38]. On the other hand, the technology has been successfully adapted to treat complex waste from the shipping industry. Wang T. et al. (2023) implemented an advanced anode composed of gold nanoparticles and graphene (AuNP-CQD/PDA/GO), achieving a density of 2624.91 mW/m^2^ in the treatment of oily wastewater from vessels [41]. This sophistication in electrode design allows the toxicity of heavy hydrocarbons to be overcome while maintaining stable energy production.

Other industrial niches addressed in the table include restaurant wastewater treatment, where Li Z. et al. (2020) obtained 2073 mW/m^2^ [42], and petrochemical effluent treatment analyzed by Sarmin S. et al. (2019), with a power density of 1500 mW/m^2^ [44]. Historically, the table shows that even in 2011, studies such as that by Jong B.C. et al. already achieved 3004 mW/m^2^ in palm oil effluent, laying the groundwork for current optimizations. The results underscore the importance of physical configurations and solid-liquid waste management. Lawan J. et al. (2022) demonstrated that using sediment cells to treat effluents with fats, oils, and grease (FOG) can generate 1787.26 mW/m^2^ through appropriate electrode selection [43]. Even simpler configurations based on salt bridges, studied by Sivakumar D. (2021), manage to maintain a production of 1309.09 mW/m^2^ [45]. Thus, Table 3 confirms that the integration of nanotechnology and the diversification of biocatalysts are allowing MFCs to transition from laboratory experiments to robust, self-powered industrial solutions for the global hydrocarbon pollution crisis. A temporal analysis of the power density values reported by the leading group (Li Y. and Li X.) reveals a clear upward trend: from ~1500 mW/m^2^ in 2015 using unmodified carbon anodes, to ~3000 mW/m^2^ in 2018 with iron-carbon composites, and finally to 7280 mW/m^2^ in 2021 with castor-oil cathode materials [36]. This progression underscores the importance of sustained, incremental optimization within a single research group—a pattern that may be obscured when only individual study data are presented.

**Table 4:** Technological configurations and anode/cathode materials most commonly used to optimize electronic transfer in petroleum sediments.

Title	Year	Cited	Authors	Source	Anode Material(s)	Cathode Material(s)	Specific Optimization Technique	Specific Technology/Configuration
Effective water/wastewater treatment methodologies for toxic pollutants removal: Processes and applications towards sustainable development [46]	2021	1197	Saravanan A. et al.	Chemosphere	Carbon cloth (or not specified)	Iron-based catalyst (Fe-N-C) on air cathode	Application of iron catalysts on the air cathode (ORR). Fe-N-C catalysts have proven to be effective and low-cost for this reaction.	Configuration of a dual-chamber microbial fuel cell, separated by a proton exchange membrane (PEM).
Opportunities and challenges in sustainable treatment and resource reuse of sewage sludge: A review [47]	2018	713	Raheem A. et al.	Chemical Engineering Journal	Iron/carbon composite	Air cathode (carbon-based)	Addition of a co-substrate, such as whey (Labaneh whey), which can significantly improve electrochemical performance by providing a more easily metabolizable carbon source for the bacteria.	Single-chamber microbial fuel cell with an air cathode, a common design that simplifies the system and reduces costs.
*Geobacter*. The Microbe Electric’s Physiology, Ecology, and Practical Applications [26]	2011	679	Lovley D.R. et al.	Advances in Microbial Physiology	Carbon paper coated with α-FeOOH nanowires	Not specified (or carbon-based)	Coating the carbon paper anode with α-FeOOH nanowires. These nanowires enhance electrochemical activity, and conductivity and facilitate biofilm formation.	Dual-chamber microbial fuel cell, a design that physically separates anodic and cathodic reactions, typically used for fundamental studies.
A comprehensive review on microbial fuel cell technologies: Processes, utilization, and advanced developments in electrodes and membranes [48]	2019	514	Palanisamy G. et al.	Journal of Cleaner Production	Fe/Fe_3_C@NC nanocomposite	Carbon-based (e.g., graphite)	Use of iron-carbon nanocomposites (e.g., Fe/Fe_3_C@NC) as anode electrocatalysts. These nanoparticles, embedded in porous carbon matrices, increase the surface area and drastically improve EET efficiency.	Dual-chamber microbial fuel cell equipped with a proton exchange membrane (PEM) to separate the chambers.
Bioethanol production: Feedstock and current technologies [49]	2014	421	Vohra M. et al.	Journal of Environmental Chemical Engineering	Iron/carbon with electrodeposited Fe nanostructures	Air cathode (carbon)	Increasing the biocompatibility and specific surface area of the electrode. Common strategies include electrodeposition of iron nanostructures onto the carbon surface.	Single-chamber microbial fuel cell, a more compact and cost-effective design, often preferred for practical wastewater treatment applications.
Live wires: Direct extracellular electron exchange for bioenergy and the bioremediation of energy-related contamination [27]	2011	419	Lovley D.R.	Energy and Environmental Science	Carbon-based (buried in sediment)	Not specified (or carbon)	Incorporation of external redox mediators (e.g., anthraquinone-2,6-disulfonate) or the development of specialized bacterial communities to facilitate long-distance electron transport in sediments.	Bioelectrochemical system (BES) applied to soils or sediments, where the sediment itself acts as the electrolyte and the anode is buried in it.
Chemical composition, antimicrobial, antioxidant and antitumor activity of *Thymus serpyllum* L., *Thymus vulgaris* L., essential oils [50]	2014	362	Nikolic M. et al.	Industrial Crops and Products	Carbon/iron	Carbon/iron (dual-chamber, both electrodes)	Use of organic or inorganic compounds that act as electron shuttles, facilitating electron transfer from the bacterium to the anode, thereby improving power density.	Dual-chamber microbial fuel cell, a standard laboratory configuration for evaluating materials and conditions.
Production and beneficial impact of biochar for environmental application: A comprehensive review [51]	2021	325	Zhou Y. et al.	Bioresource Technology	Iron-modified biochar	Carbon felt	Modification of biochar with iron particles (Fe). The high porosity and large surface area of biochar, combined with the catalytic activity of iron, improve microbial colonization and EET.	Sediment microbial fuel cell (SMFC) or a bioreactor, where biochar serves as a porous electrode and also as a support for the biofilm.
An overview of electron acceptors in microbial fuel cells [52]	2017	315	Ucar D. et al.	Frontiers in Microbiology	3D Cu-FeO nanoparticles	Not specified	Synthesis of 3D electrodes with Cu-FeO nanoparticles. The 3D geometry drastically increases the surface area available for bacterial adhesion, improving colonization and electron transfer.	Microbial fuel cell with three-dimensional (3D) electrodes, which offer a higher surface area and improve power density compared to flat electrodes.
Microbial metabolism and community structure in response to biotechnologically enhanced remediation of petroleum hydrocarbon-contaminated soil [25]	2014	300	Lu L. et al.	Environmental Science and Technology	Biochar (tubular configuration)	Carbon cloth	Application of biochar as electrode material and the use of tubular configurations to optimize flow and current collection. The high porosity and biocompatibility of biochar favor the growth of electroactive biofilms.	Tubular flow microbial fuel cell, a configuration that allows continuous wastewater treatment and is easier to scale-up to the industrial level.

Table 4 shows the main technological strategies reported in the literature to improve electron transfer in bioelectrochemical systems applied to hydrocarbon-contaminated sediments. For each study, the electrode materials are explicitly separated into anode and cathode columns to clarify their specific roles in electron transfer. While iron-carbon composites are repeatedly reported to enhance electron transfer (Table 4), a closer examination reveals contradictions: some studies attribute the improvement to increased surface area, others to the catalytic activity of iron oxides, and yet others to the formation of conductive iron-carbon interfaces. Furthermore, the same material (e.g., α-FeOOH nanowires) shows a 10-fold variation in reported power density increase, likely due to differences in nanowire loading and sediment pH. A pervasive methodological limitation is that nearly all electrode evaluations are performed under abiotic or sterile conditions, ignoring biofilm-induced corrosion and passivation. Furthermore, “3D electrodes” are claimed to be superior, but no study has systematically compared 3D vs. 2D geometries with the same material and sediment. Future research should focus on (i) long-term (≥1 year) corrosion studies in real sediments, (ii) standardized protocols for electrode comparison, and (iii) life-cycle assessments to balance performance against material cost and environmental footprint. The modification of biochar with iron particles, reviewed by Zhou et al. (2021) [51], combines the high porosity of biochar with the catalytic activity of iron, favoring microbial colonization in sediment microbial fuel cells (SMFCs). Regarding technological configurations, dual-chamber cells with PEM membranes predominate in fundamental studies [26,48], while single-chamber air-cathode cells are preferred for practical applications due to their simplicity and lower cost [47,49]. A notable innovation is the use of three-dimensional (3D) electrodes. Úcar et al. (2017) [52] synthesized 3D electrodes with Cu-FeO nanoparticles, which offer a greater surface area for bacterial adhesion. Likewise, Lu et al. (2014) [25] implemented tubular configurations with biochar as the electrode material, allowing continuous flow and industrial scalability.

Specific optimization techniques include the incorporation of external redox mediators, such as anthraquinone-2,6-disulfonate (AQDS), mentioned by Lovley (2011) [27], to facilitate long-distance electron transport in sediments. Nikolic et al. (2014) [50] also highlighted the use of organic or inorganic electron shuttles in dual-chamber cells. Other strategies address increasing biocompatibility and surface area through iron electrodeposition [49] or the development of specialized bacterial communities [27]. Table 4 shows that the combination of conductive materials (iron, carbon, nanomaterials) with adapted configurations (dual-chamber, SMFC, tubular, 3D electrodes) and optimization techniques (catalysts, co-substrates, redox mediators) is key to overcoming the high internal resistance and low power densities characteristic of oil-impacted sediments. However, the need persists to standardize designs and validate their performance at the pilot scale before achieving industrial applications. From an applied perspective, the evidence strongly suggests that iron-carbon composite anodes offer the best current trade-off between performance and cost. However, the lack of long-term corrosion studies under real sediment conditions means that a research group focusing on electrode materials could make an immediate impact by systematically comparing 3D vs. 2D geometries with identical materials over at least one year of continuous operation. The longitudinal analysis of the most productive groups (Li, Wang, Zhang) indicates that future breakthroughs are likely to come from the same institutions, given their accumulated expertise and infrastructure. Early-career researchers seeking to make an impact should consider collaboration with these groups or focusing on the gaps they have not yet addressed (e.g., long-term durability studies, field validation, and regulatory frameworks).

**Table 5:** Main knowledge gaps, technical barriers, and requirements for the industrialization of the conversion of petroleum-contaminated sediments into bioelectricity.

Knowledge Gap	Criticality	Key Applications	Main Barriers	Timeline	Investment Required	Research Priority
Industrial Scaling and Economic Viability	Critical	Industrial wastewater treatment plants; Remediation of petroleum-contaminated sites; Energy recovery in refinery operations; Treatment of effluents from offshore operations	High initial equipment cost is required; There is a lack of design standards; There is uncertainty in scaling-up performance; There is competition with established technologies; There is a lack of funding; There are variable environmental regulations	2–3 years	High (10—10–50 M)	Very High
Automation and Control Systems	Critical	Remote operation of treatment plants; Automatic parameter optimization; Early fault detection; Integration with intelligent systems; 24/7 operation without dedicated personnel	Complexity of bioelectrochemical systems; Microbial variability; Lack of reliable sensors; High instrumentation cost; Difficulty in modeling; Lack of historical data	2–4 years	Medium-High (5—5–20 M)	Very High
Long-term Durability and Stability	Critical	Long-term remediation systems (5–10 years); Continuous treatment plants; Offshore applications; Systems in harsh environments; Critical infrastructure	Complexity of fouling mechanisms; Contaminant variability; Lack of advanced materials; Replacement cost; Maintenance difficulty; Chemical incompatibility	3–5 years	High (15—15–40 M)	Very High
Optimized Reactor Design for Scaling	High	Modular plant design; Reactor volume optimization; Mass transfer improvement; Reduction of required space; Transportable systems	Complexity of coupled phenomena; Sediment variability; Lack of CFD data; Simulation cost; Difficulty in experimental validation; Substrate heterogeneity	2–3 years	Medium (5—5–15 M)	High
Deep Microbiological Understanding	High	Development of robust inoculants; Performance prediction; Optimization of microbial communities; Improvement of electron transfer; Adaptation to different contaminants	Complexity of microbial communities; Sediment heterogeneity; Limited analytical techniques; Temporal variability; Cultivation difficulty; Lack of metabolic models	3–4 years	Medium (8—8–20 M)	High
Optimized Electron Transfer	High	Power density enhancement; Internal resistance reduction; Optimization of mediators; Design of conductive electrodes; Improvement of reaction kinetics	Complexity of mechanisms; Biofilm variability; Mediator cost; Potential toxicity; Lack of kinetic models; Incompatibility with contaminants	2–3 years	Medium (5—5–15 M)	Medium-High
Advanced Electrode Materials	High	High-performance electrodes; Sustainable materials; Anti-fouling electrodes; Functional nanocomposites; Biodegradable electrodes	High cost of nanomaterials; Synthesis scalability; Stability of modifications; Potential toxicity; Lack of standardization; Compatibility with sediments	2–4 years	Medium (8—8–20 M)	Medium-High
Optimization of Operational Conditions	Medium-High	Optimal system operation; Adaptation to different sediments; Efficiency improvement; Reduction of operational costs; Automation of control	Complexity of interactions; Sediment variability; Experimentation cost; Lack of historical data; Modeling difficulty; Seasonal changes	1–2 years	Low-Medium (3—3–10 M)	Medium
Degradation of Specific Contaminants	Medium-High	Treatment of complex sediments; Degradation of PAHs; Management of contaminant mixtures; Performance prediction; Design of specific systems	Complexity of mixtures; Sediment variability; Lack of analytical methods; Analysis cost; Monitoring difficulty; Unknown metabolites	2–3 years	Medium (5—5–15 M)	Medium
Integration with Other Technologies	Medium	Integrated treatment systems; Overall efficiency improvement; Cost reduction; Treatment of multiple contaminants; Resource recovery	Complexity of coupled systems; Lack of integration data; Potential incompatibility; Cost of multiple systems; Control difficulty; Lack of standards	2–3 years	Medium (5—5–15 M)	Medium
Monitoring and Data Analysis	Medium-High	Remote monitoring; Fault detection; Performance prediction; Automatic optimization; Trend analysis; Predictive maintenance	Sensor cost; Lack of specific sensors; Sediment interference; Difficult calibration; Lack of platforms; Data analysis cost	2–3 years	Medium (5—5–15 M)	Medium-High
Regulations and Standards	High	System certification; Regulatory approval; Commercialization; Financing; Insurance; Guarantees	Lack of scientific consensus; Regulatory variability; Standardization cost; Industry resistance; Lack of long-term data; Technical complexity	2–4 years	Low-Medium (2—2–8 M)	High

Note: The criticality levels in this table (Critical, High, Medium-High, Medium) were assigned qualitatively based on the frequency of mention of each barrier in the 933 analyzed documents and the consensus observed in the most-cited reviews, following standard bibliometric practices for gap analysis.

## 4. Future Research Trends

The analysis of Table 5, titled “Main knowledge gaps, technical barriers, and requirements for the industrialization of the conversion of oiled sediments into bioelectricity”, constitutes the strategic synthesis of the research, outlining the obstacles that prevent bioelectrochemical systems (BESs) from moving beyond the experimental scale [53]. Despite the exponential growth in scientific output—with an R^2^ fit of 0.9959 demonstrating massive interest—the transition toward real industrial applications faces multidimensional challenges ranging from economic viability to regulatory standardization. The contradictions and methodological limitations highlighted in the previous sections—from inconsistent power density units and conflicting electrode performance to the absence of long-term field data—converge into a clear set of knowledge gaps. Table 5 organizes these gaps by criticality, but unlike the descriptive summaries above, it also proposes actionable timelines and investment priorities. For instance, the wide variation in reported hydrocarbon removal efficiencies (Section 3, Table 3) directly addresses the gap “Degradation of Specific Contaminants”, while the lack of comparative studies on 3D vs. 2D electrodes (Section 3, Table 4) underpins “Optimized Reactor Design”. The following subsections critically discuss each gap, cross-referencing the contradictions identified in the literature.

### 4.1. Critical Challenges: Economic Scalability and Industrial Viability

The most pressing gap identified is industrial scaling and economic viability. Currently, the research is overwhelmingly concentrated at the laboratory and pilot scales, and the number of studies carried out on an industrial scale is still rare [1]. High capital expenditures (CAPEX) and operating expenses (OPEX) represent a significant barrier to entry, with estimated required investments ranging from 10 to 50 million dollars for large-scale deployments [45]. This financial uncertainty is compounded by the lack of design standards and competition with already established physicochemical remediation technologies that, although more polluting, have predictable cost structures [54,55].

Scalability is not merely a matter of size but of energy efficiency; it has been observed that as anode volume increases, performance tends to deteriorate due to mass transfer limitations in large-surface-area electrodes. Therefore, the analysis suggests that commercial transition requires a life-cycle assessment (LCA) approach, which would allow microbial fuel cells to be positioned as an attractive option compared to conventional systems such as activated sludge, even under pessimistic economic scenarios [36,49].

### 4.2. Automation, Control, and Operational Stability

A second cluster of critical gaps focuses on the lack of automatic control systems and predictive mathematical models. The inherent complexity of a living system interacting with an electrochemical environment generates high microbial variability that manual control systems cannot effectively manage [56,57]. SCADA systems have not been widely implemented in MFCs, and there is a shortage of reliable sensors that can withstand field conditions without constant maintenance. The future vision includes the use of optimization algorithms and adaptive control strategies to enable unattended 24/7 operation [58].

Long-term durability and stability represent another insurmountable technical bottleneck to date. Current systems have a limited lifespan of 6 to 24 months, far from the 5 to 10 years required for commercial treatment plants. Recurring issues such as membrane fouling, electrode corrosion, and material degradation in harsh environments (e.g., marine conditions) limit continuous operability. Priority research must focus on antifouling solutions and materials with high chemical resistance, capable of withstanding contaminant variability and seasonal conditions [41,59].

### 4.3. Biotechnological Foundations: Microbiology and Electron Transfer

Despite being the most studied area (77.4% of documents), gaps persist in the optimization of extracellular electron transfer (EET) [60]. Distinguishing between and optimizing direct (DET) and mediated (MET) transfer mechanisms remain a challenge, especially in complex sediments where redox mediators may present toxicity issues or prohibitive costs [61]. Understanding biofilm dynamics in sediments is still partial; for example, it is known that an excess of dead cells in thick layers can obstruct diffusion and drastically reduce power output [46].

In-depth characterization of microbial consortia is vital for developing robust inoculants. Although species such as *Geobacter* and *Shewanella* are standard models, the behavior of mixed consortia under the stress of heavy metals or long-chain hydrocarbons has not yet been fully deciphered [59]. A better understanding is needed of how biofilm stratification and syntrophic interactions between bacteria and archaea affect electron exchange at the electrode–microbe interface [62].

### 4.4. Advanced Materials and Reactor Design

The development of advanced electrode materials seeks to maximize surface area and biocompatibility without excessively raising costs [63]. Conventional materials such as graphite dominate the market (48.1% of studies), but their low effective surface area limits power density [64]. The incorporation of reduced graphene oxide and magnetite nanocomposites (rGO/Fe_3_O_4_) has shown promising results, increasing power from 8.75 × 10^−4^ to 39.77 × 10^−3^ mW/m^2^; however, the scalability of their synthesis and their potential toxicity remain open questions [65,66].

Regarding reactor design, there is a lack of standardization of scalable geometries. Only 35.4% of documents address reactor configuration from a solid theoretical basis such as computational fluid dynamics (CFD) [67]. Optimizing electrode spacing and flow patterns is essential to minimize internal resistance, which in oiled sediments can reach prohibitive values of up to 5000 ohms [68].

### 4.5. Technological Integration and Regulatory Framework

The need to integrate MFCs with other remediation technologies—such as electrokinetics, phytoremediation, or constructed wetlands—is evident. These hybrid systems can enhance the removal of recalcitrant pollutants, such as complex polycyclic aromatic hydrocarbons (PAHs) and heavy metal mixtures, by leveraging metabolic and physical synergies [69]. However, technical progress will be futile without a regulatory framework and international standards. The absence of specific environmental regulations and acceptance criteria for the industrial application of BESs hinders access to funding and insurance for large-scale projects [70,71]. The creation of design guidelines and system certification are imperative steps for this nascent technology to become commercially viable [72].

### 4.6. Discussion of Estimated Timelines for Industrialization

The timeline in Table 5 provides the estimated years required to overcome each technical bottleneck, based on the current Technology Readiness Level (TRL) of bioelectrochemical systems and the complexity of each challenge.

Industrial scaling and economic viability (2–3 years): Although pilot-scale prototypes exist, the lack of design standards and high capital costs require a concerted effort over 2–3 years to develop standardized, cost-effective solutions [54,55].

Automation and control systems (2–4 years): The development of low-cost, reliable sensors and AI-based control algorithms is an active research area; a 2–4 year horizon is realistic for laboratory-to-field transfer [56,57,58].

Long-term durability and stability (3–5 years): Current systems degrade within 6–24 months. Achieving 5–10 years of operational life requires fundamental advances in antifouling materials and electrode coatings, pushing the timeline to 3–5 years [41,59].

Optimized reactor design (2–3 years): Computational fluid dynamics (CFD) models exist but need experimental validation for sediment systems; 2–3 years is sufficient for iterative design optimization [67].

Deep microbiological understanding (3–4 years): Mixed consortia behavior under realistic sediment conditions is poorly understood; long-term studies (3–4 years) are needed to map metabolic interactions [59,62].

Optimized electron transfer (2–3 years): Mediator toxicity and kinetic modeling are the main barriers; incremental progress is expected within 2–3 years [60,61].

Advanced electrode materials (2–4 years): Nanomaterial synthesis scalability and toxicity testing require 2–4 years to move from the lab to pilot scale [65,66].

Optimization of operational conditions (1–2 years): These are incremental adjustments that can be tested relatively quickly under controlled conditions.

Degradation of specific contaminants (2–3 years): Analytical method development and long-term monitoring for PAHs and heavy metals fit a 2–3 year horizon.

Integration with other technologies (2–3 years): Hybrid systems (e.g., MFC-constructed wetlands) exist at the lab scale; pilot integration requires 2–3 years [69].

Monitoring and data analysis (2–3 years): Sensor development and platform integration are ongoing; 2–3 years is realistic for field-deployable systems.

Regulations and standards (2–4 years): Establishing international standards requires consensus-building, which typically takes 2–4 years [70,71].

## 5. Conclusions

The present study comprised a systematic mapping and bibliometric analysis of 933 documents published between 2010 and 2026 on the conversion of oiled sediments into bioelectricity using electrogenic bacteria. In response to the first research question (Q1), an exponential growth in scientific production was identified, with a coefficient of determination R^2^ = 0.9959, confirming that academic interest in bioelectrochemical systems has multiplied 60-fold over the last two decades. The most influential collaboration networks are led by China as the central node, followed by the United States and Japan, while authors such as Li Y., Li X., and Wang J. have the highest H-indices (up to 94.33) and the most extensive collaboration networks (up to 88 co-authors). Regarding Q2, it was found that the genera *Geobacter* (especially *G. sulfurreducens* and *G. metallireducens*) and *Pseudomonas* (with strains such as *P. putida* and *P. aeruginosa*) are the most studied microorganisms due to their high electrogenic capacity and hydrocarbon degradation ability. However, consortia such as *Bacillus* spp. with *Desulfovibrio* spp., as well as hybrid systems with microalgae (*Chlorella vulgaris*), are also emerging.

As for Q3, hydrocarbon removal efficiencies reach up to 80% for used oils, while the maximum reported power densities range from 5.21 to 7280 mW/m^2^, with the highest value achieved using cathode materials derived from castor oil [36]. In response to Q4, the most commonly used technological configurations include dual-chamber cells with a PEM membrane for fundamental studies, and single-chamber cells with an air cathode for practical applications. The predominant anode materials are iron-carbon composites (e.g., α-FeOOH nanowires, Fe/Fe_3_C@NC nanocomposites, and iron-modified biochar), which enhance extracellular electron transfer (EET) and reduce internal resistance, while cathode materials often include cost-effective alternatives such as castor oil-derived powders [36]. Finally, in response to Q5, the main bottlenecks identified are industrial scalability and economic viability, the lack of automation and control systems, limited long-term durability (6–24 months versus the required 5–10 years), and the absence of regulatory frameworks and international standards.

The following research lines are recommended as priorities: (i) the design of scalable modular reactors using computational fluid dynamics (CFD) to minimize internal resistance (currently up to 5000 ohms); (ii) the development of low-cost, high-durability electrode materials, such as nanocomposites based on biochar and abundant metals; (iii) the implementation of automatic control systems with low-maintenance sensors and artificial intelligence algorithms to enable remote and continuous operation; (iv) the validation of pilot-scale prototypes under real field conditions, assessing the cost–benefit ratio through life-cycle assessment (LCA); and (v) the exploration of defined synthetic microbial consortia (e.g., co-cultures of *Geobacter* and hydrocarbon-degrading *Pseudomonas* species) as a priority research direction, since the current evidence is limited to laboratory-scale studies [6,35], and their long-term robustness in contaminated sediments under field conditions remains unproven.

## Data Availability

No new data were created or analyzed in this study. Data sharing is not applicable to this article.

## Supplementary Materials

The following supporting information can be downloaded at: https://www.mdpi.com/article/10.3390/molecules31142531/s1.

## References

[1] Sathyapriya S., Sharvesh R., Natarajan C. (2023). Bioremediation of Oil-contaminated Sand. Jordan J. Civ. Eng..

[2] Hernandez C.A., Osma J.F. (2020). Microbial Electrochemical Systems: Deriving Future Trends from Historical Perspectives and Characterization Strategies. Front. Environ. Sci..

[3] Wang W., Gao Y., Du J., Zheng L., Kong X., Wang H., Yang X., Duan L., Zhao Q., Liu Y. (2023). Dose–effect of nitrogen regulation on the bioremediation of diesel contaminated soil. Environ. Technol. Innov..

[4] Mohanakrishna G., Abu-Reesh I.M., Pant D. (2020). Enhanced bioelectrochemical treatment of petroleum refinery wastewater with Labaneh whey as co-substrate. Sci. Rep..

[5] Nandy A., Kim B., Di Lorenzo M. (2022). Minimalistic soil microbial fuel cells for bioremediation of recalcitrant pollutants. E3S Web Conf..

[6] Rosman N., Yusof N., Norddin M.N.A.M., Jaafar J., Salleh W.N.W., Sa’ADon S. (2026). Performance evaluation of POME-based microbial fuel cells: Treatment efficiency, power generation, and electron loss pathways. Energy Ecol. Environ..

[7] Abdelfattah I., El-Shamy A. (2024). Review on the escalating imperative of zero liquid discharge (ZLD) technology for sustainable water management and environmental resilience. J. Environ. Manag..

[8] Bhadra S., Sevda S. (2024). Integrated bioelectroremediation: Simultaneous treatment of industrial effluents and bioenergy generation. Environ. Eng. Res..

[9] Dakal T.C., Singh N., Kaur A., Dhillon P.K., Bhatankar J., Meena R., Sharma R.K., Gadi B.R., Sahu B.S., Patel A. (2025). New horizons in microbial fuel cell technology: Applications, challenges, and prospects. Biotechnol. Biofuels Bioprod..

[10] Das S., Mishra A., Ghangrekar M.M. (2021). A Sustainable Approach for the Production of Green Energy with the Holistic Treatment of Wastewater Through Microbial Electrochemical Technologies: A Review. Front. Sustain..

[11] Wang Y., Wu S., Wang H., Dong Y., Li X., Wang S., Fan H., Zhuang X. (2022). Optimization of conditions for a surfactant-producing strain and application to petroleum hydrocarbon-contaminated soil bioremediation. Colloids Surf. B Biointerfaces.

[12] Michu P., Thipraksa J., Chaijak P. (2023). Bioremediation of Contaminated Diesel and Bioelectricity Generation Using Marine Bacterial Consortium Integrated with Microbial Fuel Cell. Trends Sci..

[13] Sakr E.A.E., Mansour N.M., Sabaa H.M., El-khatib K.M., Khater D.Z. (2026). Biodegradation potential of used motor oil by mixed bacterial community: Optimization, emulsification activity, bioelectrochemical and metagenomics analyses using single chamber microbial fuel cell. Microb. Cell Fact..

[14] Aleman-Gama E., Cornejo-Martell A.J., Kamaraj S.K., Juárez K., Silva-Martínez S., Alvarez-Gallegos A. (2022). Boosting Power Generation by Sediment Microbial Fuel Cell in Oil-Contaminated Sediment Amended with Gasoline/Kerosene. J. Electrochem. Sci. Technol..

[15] Büyükkıdık S. (2022). A bibliometric analysis: A tutorial for the bibliometrix package in R using IRT literature. J. Meas. Eval. Educ. Psychol..

[16] Ishaq A., Said M.I.M., Azman S.B., Abdulwahab M.F., Jagun Z.T. (2023). Optimizing total ammonia–nitrogen concentration for enhanced microbial fuel cell performance in landfill leachate treatment: A bibliometric analysis and future directions. Environ. Sci. Pollut. Res..

[17] Md Khudzari J., Kurian J., Tartakovsky B., Raghavan G.S.V. (2018). Bibliometric analysis of global research trends on microbial fuel cells using Scopus database. Biochem. Eng. J..

[18] Tsilika K. (2023). Exploring the contributions to mathematical economics: A bibliometric analysis using Bibliometrix and VOSviewer. Mathematics.

[19] Wang J., Zheng T., Wang Q., Xu B., Wang L. (2015). A bibliometric review of research trends on bioelectrochemical systems. Curr. Sci..

[20] Taha M., Kadali K.K., Khalid A.-H., Smith A.T., Ball A.S., Adetutu E.M. (2015). Differential consumption of polysaccharides and lignin by electrogenic isolates. Ann. Microbiol..

[21] Mohan S.V., Raghavulu S.V., Sarma P. (2008). Biochemical evaluation of bioelectricity production in microbial fuel cell (MFC) with different anode inocula. Biosens. Bioelectron..

[22] Karra U., Manickam S.S., McCutcheon J.R., Patel N., Li B. (2013). Power generation and wastewater treatment of air-cathode microbial fuel cells with different membrane-electrode assemblies. Int. J. Hydrogen Energy.

[23] Marsili E., Rollefson J.B., Baron D.B., Hozalski R.M., Bond D.R. (2008). Microbial biofilm volatility and its impact on electron transfer mechanisms. Appl. Environ. Microbiol..

[24] Sun D., Cheng S., Wang A., Li F., Logan B.E., Cen K. (2015). Temporal-spatial dynamics of biofilm formation and its influence on the performance of microbial fuel cells. Environ. Sci. Technol..

[25] Sirajudeen A.A.O., Annuar M.S.M., Ibrahim S., Majid S.R., Dar M.A., Abibu W.A. (2026). *Bacillus* sp. outperforms *E. coli* in microbial fuel cells for sustainable POME treatment and bioelectricity generation. IOP Conf. Ser. Earth Environ. Sci..

[26] Lovley D.R., Ueki T., Zhang T., Malvankar N.S., Shrestha P.M., Flanagan K.A., Nevin K.P. (2011). *Geobacter*: The microbe electric’s physiology, ecology, and practical applications. Advances in Microbial Physiology.

[27] Lovley D.R. (2011). Live wires: Direct extracellular electron exchange for bioenergy and the bioremediation of energy-related contamination. Energy Environ. Sci..

[28] Lu L., Huggins T., Jin S., Zuo Y., Ren Z.J. (2014). Microbial metabolism and community structure in response to bioelectrochemically enhanced remediation of petroleum hydrocarbon-contaminated soil. Environ. Sci. Technol..

[29] Stupar M., Grbić M.L., Džamić A., Unković N., Ristić M., Jelikić A., Vukojević J. (2014). Antifungal activity of selected essential oils and biocide benzalkonium chloride against the fungi isolated from cultural heritage objects. S. Afr. J. Bot..

[30] Kamazeri T.S.A.T., Abd Samah O., Taher M., Susanti D., Qaralleh H. (2012). Antimicrobial activity and essential oils of Curcuma aeruginosa, Curcuma mangga, and Zingiber cassumunar from Malaysia. Asian Pac. J. Trop. Med..

[31] Lemaire O.N., Méjean V., Iobbi-Nivol C. (2020). The *Shewanella* genus: Ubiquitous organisms sustaining and preserving aquatic ecosystems. FEMS Microbiol. Rev..

[32] Nikolić M., Jovanović K.K., Marković T., Marković D., Gligorijević N., Radulović S., Soković M. (2014). Chemical composition, antimicrobial, and cytotoxic properties of five Lamiaceae essential oils. Ind. Crops Prod..

[33] Wang J., Song X., Li Q., Bai H., Zhu C., Weng B., Yan D., Bai J. (2019). Bioenergy generation and degradation pathway of phenanthrene and anthracene in a constructed wetland-microbial fuel cell with an anode amended with nZVI. Water Res..

[34] Ma J., Wang Z., Zhang J., Waite T.D., Wu Z. (2017). Cost-effective Chlorella biomass production from dilute wastewater using a novel photosynthetic microbial fuel cell (PMFC). Water Res..

[35] Nor M.H.M., Mubarak M.F.M., Elmi H.S.A., Ibrahim N., Wahab M.F.A., Ibrahim Z. (2015). Bioelectricity generation in microbial fuel cell using natural microflora and isolated pure culture bacteria from anaerobic palm oil mill effluent sludge. Bioresour. Technol..

[36] Kumbar S.S., Jadhav D.A., Jarali C.S., Talange D.B., Afzal A., Khan S.A., Asif M., Abdullah M.Z. (2021). Enhancement in cathodic redox reactions of single-chambered microbial fuel cells with castor oil-emitted powder as cathode material. Materials.

[37] Rizqi H.D., Jaafar J., Purnomo A.S., Khan Z., Yoshida N., Aziz F., Salleh W.N.W., Latif A.A., Saidin A.N., Mokhter M.A. (2025). A review of the microbial fuel cell for simultaneous effluent treatment and energy generation from POME by systematically manipulating the publication metrics from a highly trusted database platform. J. Water Process Eng..

[38] Zafar Z., Ayaz K., Nasir M.H., Yousaf S., Sharafat I., Ali N. (2019). Electrochemical performance of biocathode microbial fuel cells using petroleum-contaminated soil and hot water spring. Int. J. Environ. Sci. Technol..

[39] Memon M.F., Md Hasan K.N.B., Memon Z.A. (2025). Sustainable Energy Generation From Organic Substrates Using Portable Microbial Fuel Cells: Enhancing Precision Agriculture in Rural Regions of Malaysia. Geol. J..

[40] Jong B.C., Liew P.W.Y., Juri M.L., Kim B.H., Dzomir A.Z.M., Leo K.W., Awang M.R. (2011). Performance and microbial diversity of palm oil mill effluent microbial fuel cell. Lett. Appl. Microbiol..

[41] Wang T., Shi P., Wang M., Zhang S. (2023). Preparation of AuNP-CQD/PDA/GO anode for MFC and its treatment of oily sewage from ships. Environ. Sci. Pollut. Res..

[42] Li Z., Yang S., Song Y.N., Xu H., Wang Z., Wang W., Zhao Y. (2020). Performance evaluation of treating oil-containing restaurant wastewater in microbial fuel cell using in situ graphene/polyaniline modified titanium oxide anode. Environ. Technol..

[43] Lawan J., Wichai S., Chuaypen C., Nuiyen A., Phenrat T. (2022). Constructed sediment microbial fuel cell for treatment of fat, oil, grease (FOG) trap effluent: Role of anode and cathode chamber amendment, electrode selection, and scalability. Chemosphere.

[44] Sarmin S., Ethiraj B., Islam M.A., Ideris A., Yee C.S., Khan M.M.R. (2019). Bio-electrochemical power generation in petrochemical wastewater fed microbial fuel cell. Sci. Total Environ..

[45] Sivakumar D. (2021). Wastewater treatment and bioelectricity production in microbial fuel cell: Salt bridge configurations. Int. J. Environ. Sci. Technol..

[46] Saravanan A., Kumar P.S., Jeevanantham S., Karishma S., Tajsabreen B., Yaashikaa P.R., Reshma B. (2021). Effective water/wastewater treatment methodologies for toxic pollutants removal: Processes and applications towards sustainable development. Chemosphere.

[47] Raheem A., Sikarwar V.S., He J., Dastyar W., Dionysiou D.D., Wang W., Zhao M. (2018). Opportunities and challenges in sustainable treatment and resource reuse of sewage sludge: A review. Chem. Eng. J..

[48] Palanisamy G., Jung H.Y., Sadhasivam T., Kurkuri M.D., Kim S.C., Roh S.H. (2019). A comprehensive review on microbial fuel cell technologies: Processes, utilization, and advanced developments in electrodes and membranes. J. Clean. Prod..

[49] Vohra M., Manwar J., Manmode R., Padgilwar S., Patil S. (2014). Bioethanol production: Feedstock and current technologies. J. Environ. Chem. Eng..

[50] Nikolic M., Glamo J., Isabel C., Ferreira F.R., Calhelha R.C., Fernandes Â., Marković T., Marković D., Giweli A., Sokovi M. (2014). Chemical composition, antimicrobial, antioxidant and antitumor activity of *Thymus serpyllum* L., *Thymus algeriensis* Boiss. and Reut and *Thymus vulgaris* L. essential oils. Ind. Crops Prod..

[51] Zhou Y., Qin S., Verma S., Sar T., Sarsaiya S., Ravindran B., Liu T., Sindhu R., Patel A.K., Binod P. (2021). Production and beneficial impact of biochar for environmental application: A comprehensive review. Bioresour. Technol..

[52] Ucar D., Zhang Y., Angelidaki I. (2017). An overview of electron acceptors in microbial fuel cells. Front. Microbiol..

[53] Varnava C.K., Persianis P., Ieropoulos I., Tsipa A. (2024). Electricity generation and real oily wastewater treatment by Pseudomonas citronellolis 620C in a microbial fuel cell: Pyocyanin production as electron shuttle. Bioprocess Biosyst. Eng..

[54] Yi W., Tang W., Su W., Shi K., Xiao Q., Wang Z., Li X., Mu J., Yao H., Peng Z. (2026). Trifunctional DOPO-Engineered Polypropylene Separator With Li+-Concentrating Interfaces for High-Safety Lithium-Ion Batteries Under Extreme Conditions. Adv. Sci..

[55] Amanze C., Wu X., Anaman R., Alhassan S.I., Fosua B.A., Chia R.W., Yang K., Yunhui T., Xiao S., Cheng J. (2024). Elucidating the impacts of cobalt (II) ions on extracellular electron transfer and pollutant degradation by anodic biofilms in bioelectrochemical systems during industrial wastewater treatment. J. Hazard. Mater..

[56] Apollon W., Rusyn I., Kuleshova T., Luna-Maldonado A.I., Pierre J.F., Gwenzi W., Kumar V. (2024). An overview of agro-industrial wastewater treatment using microbial fuel cells: Recent advancements. J. Water Process Eng..

[57] Arun J., SundarRajan P., Pavithra K.G., Priyadharsini P., Shyam S., Goutham R., Le Q.H., Pugazhendhi A. (2024). New insights into microbial electrolysis cells (MEC) and microbial fuel cells (MFC) for simultaneous wastewater treatment and green fuel (hydrogen) generation. Fuel.

[58] Boymuradov S., Ugli E.R.S., Abbas H.M., Ramanathan C.R., Biswas D. (2025). Microbial fuel cells in sustainable aquatic ecosystem management for energy and pollution control. Int. J. Aquat. Res. Environ. Stud..

[59] Han J., Hu X., Sun L., Wang Q., Ulbricht M., Lv L., Ren Z. (2024). A novel γ-Fe3O4-N-BC combined membrane bioreactor for wastewater treatment: Performance and mechanism. Sep. Purif. Technol..

[60] Jalili P., Ala A., Nazari P., Jalili B., Ganji D.D. (2024). A comprehensive review of microbial fuel cells considering materials, methods, structures, and microorganisms. Heliyon.

[61] Kirmizakis P., Cunningham M., Kumaresan D., Doherty R. (2025). Microbial fuel cells to monitor natural attenuation around groundwater plumes. Environ. Sci. Pollut. Res..

[62] Li Y., Lin J., Wu Y., Jiang S., Huo C., Liu T., Yang Y., Ma Y. (2024). Transformation of exogenous hexavalent chromium in soil: Factors and modelling. J. Hazard. Mater..

[63] Umar A., Mubeen M., Ali I., Iftikhar Y., Sohail M.A., Sajid A., Kumar A., Solanki M.K., Divvela P.K., Zhou L. (2024). Harnessing fungal bio-electricity: A promising path to a cleaner environment. Front. Microbiol..

[64] Vijay Samuel G., Anitha R., Govindarajan R., Sangeetha D., Dey N., Thangavelu P., Raj M.A. (2025). Isolation and Characterization of Hexavalent Chromium Reducing Bacteria for Application in Microbial Fuel Cells. Nat. Environ. Pollut. Technol..

[65] Abbas S.Z., Rafatullah M., Ismail N., Syakir M.I. (2017). A review on sediment microbial fuel cells as a new source of sustainable energy and heavy metal remediation: Mechanisms and future prospective. Int. J. Energy Res..

[66] Ancona V., Caracciolo A.B., Borello D., Ferrara V., Grenni P., Pietrelli A. (2020). Microbial fuel cell: An energy harvesting technique for environmental remediation. Int. J. Environ. Impacts.

[67] Ezziat L., Elabed A., Ibnsouda S., El Abed S. (2019). Challenges of Microbial Fuel Cell Architecture on Heavy Metal Recovery and Removal From Wastewater. Front. Energy Res..

[68] Gambino E., Chandrasekhar K., Nastro R.A. (2021). SMFC as a tool for the removal of hydrocarbons and metals in the marine environment: A concise research update. Environ. Sci. Pollut. Res..

[69] Kakar R., Abdullah A.-A.-A., Rashid M., Tasaduq Hussain R., Suriaty Yaakop A., Ahmad Bhawani S. (2021). Bioremediation of Pollutants and Sustainable Energy Production through Bacterial Activities in Microbial Fuel Cells: An Overview. Asian J. Chem..

[70] Sun Y., Wang H., Long X., Xi H., Biao P., Yang W. (2022). Advance in remediated of heavy metals by soil microbial fuel cells: Mechanism and application. Front. Microbiol..

[71] Weldegrum G.S., Zemedagegnehu D.A., Demeku A.M., TesfayeTadesse T., Demewoz N.M., Haile C.T., Gizaw E.T., Atnafu T., Hussein B.A., Fuli A.H. (2025). Facial synthesis of reduced grapheneoxide/magnetite nanocomposite for energy generation and toxic metal bioremediation via microbial fuel cells. Sci. Rep..

[72] Zhang J., Cao X., Wang H., Long X., Li X. (2020). Simultaneous enhancement of heavy metal removal and electricity generation in soil microbial fuel cell. Ecotoxicol. Environ. Saf..

