# Supplementary material for: A Bibliometric Analysis of Redox Species and Bio-Derived Electrodes for the Conversion of Petroleum-Contaminated Sediments into Bioelectricity
Source: Molecules. 2026 Jul 21;31(14):2531. doi: 10.3390/molecules31142531 (PMC13415048; doi:10.3390/molecules31142531)
Supplement: Supplementary file 1 [file molecules-31-02531-s001.zip › molecules-4364949-supplementary.pdf]

Supplementary Table S1

Thematic area	Most active countries	Leading institutions (examples)	Representative reference(s)
Agricultural sediments	China, USA, Japan	Nankai University, Chinese Academy of Sciences, USDA-ARS	Wang et al., 2019 [33]; Lu et al., 2014 [28]
Refinery sediments	China, Qatar, India	Chongqing University, Qatar University, Indian Institute of Technology	Mohanakrishna et al., 2020 [4]; Aleman-Gama et al., 2022 [14]
Palm oil pollution	Thailand, Malaysia, Indonesia	Thaksin University, Universiti Sains Malaysia, Bogor Agricultural University	Michu et al., 2023 [12]; Nor et al., 2015 [35]
EET mechanisms	USA, China, Japan	University of Massachusetts Amherst, Nankai University, University of Tokyo	Lovley et al., 2011 [26]; Lovley, 2011 [27]; Marsili et al., 2008 [23]
Biofilms	USA, China, Germany	University of Southern California, Nankai University, Max Planck Institute	Sun et al., 2015 [24]; Marsili et al., 2008 [23]; Han et al., 2024 [59]
